# A molecular comparative study of intestinal colonization with *Staphylococcus aureus* between pediatric inpatients and outpatients of different age groups

**DOI:** 10.1128/spectrum.02394-24

**Published:** 2025-05-16

**Authors:** Shaoxiang Lin, Zhile Xiong, Chao Zhang, Shuyan Liu, Tongyan Ding, Kaiyue Yang, Yunxing He, Zhimin Zhao, Zhenwen Zhou

**Affiliations:** 1 Affiliated Shenzhen Women and Children's Hospital (Longgang) of Shantou University Medical College (Longgang District Maternity & Child Healthcare Hospital of Shenzhen City), Medical Research Institute of Maternal and Child, Shenzhen, China; 2 Charité – Universitätsmedizin Berlin Corporate Member of Freie Universität Berlin and Humboldt- Universität zu Berlin, Institute of Microbiology, Infectious Diseases and Immunology, Berlin, Germany; University at Albany, Albany, New York, USA

**Keywords:** *Staphylococcus aureus*, *S. aureus*, MRSA, children, multilocus sequence typing, MLST, Shenzhen

## Abstract

Currently, *Staphylococcus aureus* (*S. aureus*) is one of the leading causes of death from infectious diseases worldwide. Our aim in this study was to investigate and compare the molecular epidemiology and antibiotic resistance of *S. aureus* colonizing the intestinal tract of pediatric inpatients and outpatients of different age groups. We analyzed stool samples from 1,300 patients at a children’s hospital in Shenzhen, China. After culturing *S. aureus*, we used matrix-assisted laser desorption/ionization time-of-flight mass spectrometry to identify the strains. In addition, we performed antimicrobial susceptibility testing on isolated *S. aureus* strains as well as pheno- and genotypic characterization by PCR. *S. aureus* was detected in 104 out of 1,300 (8.0%) patients, including 20 out of 1,300 (1.5%) patients with methicillin-resistant *S. aureus* (MRSA). MRSA accounted for 19.2% of the *S. aureus* isolates. The resistance rates of *S. aureus* strains to penicillin, erythromycin, clindamycin, levofloxacin, and moxifloxacin were 83.7%, 34.6%, 31.7%, 3.9%, and 3.9%, respectively. None of the strains showed resistance to linezolid, daptomycin, tigecycline, vancomycin, or tetracycline. One hundred fou strains of *S. aureus* revealed that 49.0% (51/104) harbored enterotoxin genes, and most enterotoxin-positive strains carried only one gene type (90.2%, 46/51), while a minority carried two gene types (9.8%, 5/51). Besides, a total of 29 sequence types (STs) were identified with the three most prevalent STs: ST45, ST188, and ST6, accounting for 12.5%, 12.5%, and 9.6%. We found that the molecular characteristics of *S. aureus* in intestinal colonization of children have regional differences. To provide a theoretical basis for the prevention and control of *S. aureus* infections, increased surveillance of local *S. aureus* resistance and molecular epidemiological characteristics is needed.

**IMPORTANCE:**

This study assessed the clinical and molecular epidemiology of *Staphylococcus aureus* in pediatric patients at a children’s hospital in Shenzhen, South China by means of screening stool samples for pheno- and genotypic characterization for carriage of *S. aureus*. Of 1,300 fecal samples screened, 104 (8.0%) were positive for *S. aureus* with 19.2% methicillin-resistant *S. aureus*. The resistance rates of *S. aureus* to penicillin, erythromycin, clindamycin, levofloxacin, and moxifloxacin were 83.7%, 34.6%, 31.7%, 3.9%, and 3.9%, respectively. None of the strains showed resistance to linezolid, daptomycin, tigecycline, vancomycin, or tetracycline. One hundred four strains of *S. aureus* revealed that 49.0% (51/104) harbored enterotoxin genes, and most enterotoxin-positive strains carried only one gene type (90.2%, 46/51), while a minority carried two gene types (9.8%, 5/51). Besides, a total of 29 sequence types (STs) were identified with the three most prevalent STs: ST45, ST188, and ST6, accounting for 12.5%, 12.5%, and 9.6%.

## INTRODUCTION

The gram-positive bacterium *Staphylococcus aureus* (*S. aureus*) is responsible for a variety of infectious diseases. Not only is it one of the main causes of bacteremia and infective endocarditis, but it can also cause infections of the skin, respiratory tract, bones and joints, abdomen or urinary tract (1, 2). Currently*, S. aureus* remains one of the leading causes of death from infectious diseases worldwide, with over 1 million deaths per year (1). The increasing global prevalence of methicillin-resistant *S. aureus* (MRSA) represents an ongoing public health challenge and significantly complicates treatment (3). Alternative treatment and prevention measures such as vaccines are currently under development (3).

The ability of *S. aureus* to cause a broad range of infections is mainly attributed to its diverse spectrum of virulence factors (4). Regardless of antibiotic resistance, *S. aureus* has the ability to produce various toxins and enzymes to increase its virulence. One example is that *S. aureus* strains produce Panton-Valentine leukocidin (PVL). PVL is a toxin that creates pores in leukocytes, especially granulocytes, monocytes, and macrophages, thereby lysing them (5). PVL-positive *S. aureus* is associated with severe infections such as necrotizing pneumonia and frequently causes recurrent skin and soft tissue infections. *S. aureus* is known to asymptomatically colonize human epithelial and mucosal surfaces, such as the skin, nose, throat, intestine, or vagina (6). Colonization frequency may vary depending on geographic location and socioeconomic factors (7). Asymptomatic colonization with *S. aureus* may lead to infection and transmission to others, especially in the hospital (6). Therefore, decolonization plays a key role in infection prevention. Currently, most decolonization treatments focus on eradicating *S. aureus* in the nose and on the skin with antibiotics or antiseptics. However, recent research shows that intestinal colonization may represent the key reservoir for *S. aureus* (6). Screening by examining stool samples can effectively identify patients whose intestines are colonized by *S. aureus* (8). Molecular characterization of isolated *S. aureus* strains supports the prevention and monitoring of the spread of *S. aureus* (9).

Several studies have investigated the genetic distribution of *S. aureus*, revealing different patterns in different regions. For example, the ST239-MRSA-IIIA genotype is predominant in several Asian and South American countries (10, 11), while the ST5-MRSA-II genotype is predominant in certain Western countries (12). Further genotyping studies of MRSA strains isolated from pediatric patients in China have identified predominant genotypes in different cities, such as ST59-MRSA-IVa in Guangzhou, Beijing, and Chongqing or ST239-MRSA-III in Shanghai (13–15). Understanding the epidemiology and origin of *S. aureus* is critical to developing effective public health measures to contain its spread both locally and globally. Our aim in this study was to investigate and compare the molecular epidemiology and antibiotic resistance of *S. aureus* colonizing the intestinal tract of pediatric inpatients and outpatients of different age groups.

## MATERIALS AND METHODS

### Sampling and study population

This comparative study was conducted in Shenzhen Children’s Hospital and Longgang District Maternity & Child Healthcare Hospital, Shenzhen, China. We collected stool samples from a total of 1,300 patients, including 538 outpatients and 762 inpatients, between July and September 2022. Clinical data, including age, sex, and medical history, were collected from the hospital information system. For analysis, patients were divided into six age groups: newborns (0–28 days), infants (29 days to 6 months), toddlers (6 months to 1 year), young children (1–3 years), preschoolers (3–6 years), and school-aged children (6–12 years).

### Strain identification and antimicrobial susceptibility testing

Stool samples were individually inoculated onto mannitol salt agar plates and incubated for 48 h at 35°C with 5% CO_2_. Based on colony growth morphology and coagulase tests, suspected *S. aureus* colonies were subcultured onto Columbia blood agar plates and incubated overnight at 35°C with 5% CO_2_.

Strains were identified using matrix-assisted laser desorption/ionization time-of-flight mass spectrometry (Bruker Corporation, Billerica, MA, USA). Antibiotic susceptibility testing for isolated *S. aureus* was performed using a Vitek 2 Compact system (bioMérieux) with GP67 drug susceptibility cards according to the manufacturer’s instructions. The susceptibility of each strain to 15 antibiotics (penicillin (≤0.12 µg/mL), oxacillin (≤2 µg/mL), ceftaroline (≤1 µg/mL), gentamicin (≤4 µg/mL), levofloxacin (≤1 µg/mL), erythromycin (≤0.5 µg/mL), clindamycin (≤0.5 µg/mL), linezolid (≤4 µg/mL), daptomycin (≤1 µg/mL), teicoplanin (≤8 µg/mL), vancomycin (≤2 µg/mL), tigecycline (≤2 µg/mL), rifampicin (≤1 µg/mL), and trimethoprim/sulfamethoxazole (≤2/38 µg/mL) was determined using VITEK 2 AST-GP67 cards (bioMérieux). All antimicrobial results are according to Clinical and Laboratory Standards Institute Performance Standards for Antimicrobial Susceptibility Testing, M100 (16). Each strain was independently obtained and maintained as a stock culture at −80°C in Lysogeny broth supplemented with 20% glycerol until further analysis.

### Genotyping

To detect the mecA and PVL gene as well as enterotoxin genes by gel electrophoresis, bacterial DNA was extracted from isolated *S. aureus* strains using a commercially available DNA Extraction Kit (Accurate Biology, Hunan, China) according to the manufacturer’s instructions. And then amplified by polymerase chain reaction (PCR), using the primers 5′-ATCATTAGGTAAAATGTCTTGGACATGATCC-3′; 5′-GCATCAACTGTATTGGATAGCAAAAGC-3′ for PVL genes 18–19 (lukS/F-PV) and 5′-GTAGAAATGACTGAACGTCCGATAA-3′; 5′-CCAATTCCACATTGTTTCGGTCTAA-3′ for the mecA gene, as described before (8, 17).

### SCCmec typing

A multiplex PCR was used to determine the specific type of staphylococcal cassette chromosome mec (SCCmec). The results were then classified into types I–V. Isolates that did not fit into types I–V were classified as non-typeable (NT).

### Multilocus sequence typing (MLST)

For MLST, seven housekeeping genes (arcC, aroE, glpF, gmK, pta, tpi, and yqi) were amplified by PCR with primers recommended by the MLST databases listed below and subsequently detected by gel electrophoresis (18). The sequence types (STs) and clone complexes (CCs) of each strain were determined by comparison with known alleles in the mlst.net and PubMLST databases. We used PHYLOViZ Online (http://www.phyloviz.net) to construct a dendrogram of the STs and assess the evolutionary relationship among the isolates.

### Statistical analysis

Statistical analysis was conducted using IBM SPSS (Version 22.7). Comparisons of categorical data between gender groups were assessed using Fisher’s exact test, with a significance threshold of *P* < 0.05. Comparisons of categorical data across department groups, enterotoxins between MRSA and MSSA, and antibiotic profiles between MRSA and MSSA or ST types were evaluated using the chi-square test for Independence, with a significance threshold of *P* < 0.05. Additionally, comparisons of categorical data within age groups were assessed using the chi-square for trend test, with a significance threshold of *P* < 0.05.

## RESULTS

### Prevalence of intestinal colonization with *S. aureus*

A total of 1,300 pediatric patients, including 538 outpatients and 762 inpatients, were screened for intestinal colonization with *S. aureus* (Table 1). The presence of *S. aureus* was detected in 104 out of 1,300 (8.0%) patients, including 20 out of 1,300 (1.5%) patients with MRSA (Tables 1 and 2). MRSA accounted for 19.2% of the *S. aureus* isolates. The positivity rate for *S. aureus* was significantly higher in outpatients (11.3%) than in inpatients (5.6%). The detection rate of *S. aureus* in newborns (0–28 days) was 3.9%. The chi-square for trend test shows that in the five other age groups ranging from 29 days to 12 years, the colonization rate of intestinal *S. aureus* decreased with increasing age, showing a significant negative correlation with age (*χ*² = 4.543, *P* = 0.03) (Table 1). Subsequently, in Table 2, we found that there was no significant correlation between the MRSA positive rate and gender, department, or age (*P* > 0.05).

**TABLE 1:** Correlation of *S. aureus* in pediatric patients’ fecal samples with different clinical features

Variable	Group	Total no. of patients (*n* = 1,300)	*S. aureus* (*n* = 104)	*χ* ^2^	*P* value
Gender				0 (Fisher’s exact test)	0.97
	Males	801	65 (8.1%)		
	Females	499	39 (7.8%)		
Department				22.84 (chi-square for independence test)	<0.001
	Pediatrics (outpatients)	305	41 (13.4%)		
	Pediatric emergency (outpatients)	233	19 (8.2%)		
	Pediatrics (inpatients)	354	28 (7.9%)		
	Neonatology (inpatients)	408	15 (3.7%)		
Age					
	0–28 days	408	16 (3.9%)		
				4.543 (chi-square for trend test)	0.03
	28 days to 6 months	202	28 (13.9%)		
	6 months to 1year	353	36 (10.2%)		
	1–3 years	166	11 (6.6%)		
	3–6 years	112	8 (7.1%)		
	6–12 years	59	5 (8.5%)		

**TABLE 2:** Correlation of MRSA in pediatric patients’ fecal samples with different clinical features

Variable	Group	Total no. of patients (*n* = 1,300)	MRSA (*n* = 20)	*χ* ^2^	*P* value
Gender				0.604 (Fisher’s exact test)	0.437
	Males	801	14 (1.8%)		
	Females	499	6 (1.2%)		
Department				7.215 (chi-square for independence test)	0.06
	Pediatrics (outpatients)	305	8 (2.6%)		
	Pediatric emergency (outpatients)	233	1 (0.6%)		
	Pediatrics (inpatients)	354	8 (2.3%)		
	Neonatology (inpatients)	408	3 (0.7%)		
Age				2.747 (chi-square for trend test)	0.097
	0–28 days	408	2 (0.5%)		
	28 days to 6 months	202	4 (2.0%)		
	6 months to 1year	353	8 (2.3%)		
	1–3 years	166	1 (0.6%)		
	3–6 years	112	4 (3.6%)		
	6–12 years	59	1 (1.7%)		

### Antimicrobial resistance testing

As shown in Table 3, the resistance rates of *S. aureus* strains to penicillin, erythromycin, clindamycin, levofloxacin, moxifloxacin, and linezolid were 83.7%, 34.6%, 31.7%, 3.9%, 3.9%, and 3.9%, respectively. None of the strains showed resistance to linezolid, daptomycin, tigecycline, vancomycin, or tetracycline. MRSA strains showed higher resistance rates to penicillin (100.0%), erythromycin (65.0%), and clindamycin (60.0%) compared to MSSA strains to penicillin (79.8%), erythromycin (27.4%), and clindamycin (25.0%) (Table 3).

**TABLE 3:** Antibiotic profiles of *S. aureus* isolated from pediatric patients’ fecal samples^*a*^

	*S. aureus*		MSSA		MRSA		*χ*^2^ (*P* value)
	*R*, *n* (%)	*S*, *n* (%)	*R*, *n* (%)	*S*, *n* (%)	*R*, *n* (%)	*S*, *n* (%)	
Penicillin	87 (83.65%)	17 (16.35%)	67 (79.76%)	17 (20.33%)	20 (100%)	0 (0%)	4.839 (*P* = 0.0278)
Ceftaroline	0 (0%)	104 (100%)	0 (0%)	84 (100%)	0 (0%)	20 (100%)	NA
Gentamycin	2 (1.92%)	100 (96.15%)	2 (3.38%)	80 (95.24%)	0 (0%)	20 (100%)	0.486 (*P* = 0.4855)
Levofloxacin	4 (3.85%)	97 (93.27%)	3 (3.57%)	79 (94.05%)	1 (5.00%)	19 (95.00%)	0.089 (*P* = 0.7653)
Moxifloxacin	4 (3.85%)	100 (96.15%)	3 (3.57%)	81 (96.43%)	1 (5.00%)	19 (95.00%)	0.089 (*P* = 0.7653)
Erythromycin	36 (34.62%)	68 (65.38%)	23 (27.38%)	61 (72.62%)	13 (65.00%)	7 (35.00%)	10.1 (*P* = 0.0015)
Clindamycin	33 (31.73%)	71 (68.27%)	21 (25.00%)	63 (75%)	12 (60.00%)	8 (40.00%)	9.135 (*P* = 0.0025)
Linezolid	0 (0%)	104 (100%)	0 (0%)	84 (100%)	0 (0%)	20 (100%)	NA
Daptomycin	0 (0%)	104 (100%)	0 (0%)	84 (100%)	0 (0%)	20 (100%)	NA
Teicoplanin	0 (0%)	104 (100%)	0 (0%)	84 (100%)	0 (0%)	20 (100%)	NA
Vancomycin	0 (0%)	104 (100%)	0 (0%)	84 (100%)	0 (0%)	20 (100%)	NA
Tigecycline	0 (0%)	104 (100%)	0 (0%)	84 (100%)	0 (0%)	20 (100%)	NA
Rifampin	3 (2.88%)	95 (91.37%)	2 (2.38%)	79 (94.05%)	1 (5.00%)	16 (80.00%)	NA
Trimethoprim/sulfamethoxazole	4 (3.85%)	100 (96.15%)	4 (4.76%)	80 (95.24%)	0 (0%)	20 (100%)	0.991 (*P* = 0.3196)

^*a*^ R, resistant; S, sensitive; NA, not applicable.

### Genotyping *mecA* gene

To confirm MRSA, PCR-based detection of the mecA gene was performed. All of the 20 MRSA isolates showed positive bands with a size of 310 bp, indicating the presence of the *mecA* gene (Table 4).

**TABLE 4:** Detection of *mecA*, *pcl* gene, and SCCmec typing in MRSA isolated from pediatric patients’ fecal samples

Gene	MRSA
mecA	20 (100%)
PVL	2 (10%)
SccmecA-Iva	13 (65%)
Total	20

### lukS and lukF-PV genes (encoding PVL)

PCR-based detection of the lukS and lukF-PV genes was performed on all 104 *S*. *aureus* strains. The lukS and lukF-PV genes were not detected in any of the 84 MSSA strains, but in 2 of the 20 MRSA strains (target band at 433 bp) (Table 4).

### Enterotoxin genes

As shown in Table 5, PCR-based analysis of all 104 strains of *S. aureus* revealed that 49.0% (51/104) harbored enterotoxin genes, with a higher prevalence of 75.0% (15/20) observed in MRSA strains. Most enterotoxin-positive strains carried only one gene type (90.2%, 46/51), while a minority carried two gene types (9.8%, 5/51). Statistical analysis showed no association between enterotoxin genes and age, sex, or the department.

**TABLE 5:** Genotyping of *S. aureus* enterotoxins along with clinical diagnostics

	*sea*	*seb*	*sec*	*sea* + *seb*	*sea* + *sec*	*sec* + *sed*	Non enterotoxin carrier	Total (%)
Gastroenteritis	7	4	5	1	0	1	28	46 (44.2%)
Bronchopneumonia or pneumonia	7	3	1	0	1	0	15	27 (26.0%)
Dermatitis	1	0	0	0	0	1	0	2 (1.9%)
Neonatal hyperbilirubinemia	1	0	2	0	1	0	5	8 (7.7%)
Preterm birth	1	1	1	0	0	0	2	5 (4.8%)
Genitourinary disorders	0	1	6	0	0	0	1	8 (7.7%)
Neurological disorders	1	0	5	0	0	0	2	8 (7.7%)
Total	17	9	20	1	2	2	53	104 (100%)

Analysis of the expression of enterotoxin genes in MRSA and MSSA revealed that the *sec* was detected in 20 strains of *S. aureus* with MRSA and MSSA positivity rates of 30.00% (6/20) and 16.7% (14/84), respectively. In addition, the *sea* was found in 20 *S. aureus* strains with MRSA and MSSA positivity rates of 15.00% (3/20) and 20.23% (17/84), respectively. Furthermore, the *seb* was detected in nine *S*. *aureus* strains, with MRSA and MSSA positivity rates of 25.00% (5/20) and 6.00% (5/84), respectively. Moreover, there are two strains MSSA with *sea + sec* and two strains MSSA with *sec + sed* detected. No other gene-positive strains were detected (Table 5).

### Association of enterotoxin genes with clinical diagnostics

As shown in Table 6, seven clinical diagnostics including gastroenteritis, bronchopneumonia or pneumonia, dermatitis, neonatal hyperbilirubinemia, preterm birth, genitourinary disorders, and neurological disorders were documented in pediatric patients colonized with *S. aureus*. Among carriers of *S. aureus* with gastroenteritis, 28 strains did not carry enterotoxin genes, while 7 strains carried the *sea* gene, 4 strains carried the *seb* gene, 5 strains carried the *sec* gene, 1 strain carried both the *sea* and *sec* genes, and 1 strain carried both the *sec* and *sed* genes. Among carriers of *S. aureus* with bronchopneumonia or pneumonia, 15 strains did not carry enterotoxin genes, while 7 strains carried the *sea* gene, 3 strains carried the *seb* gene, 1 strain carried the *sec* gene, and 1 strain carried both the *sea* and *sec* genes. Among carriers of *S. aureus* with genitourinary disorders, one strain did not carry enterotoxin genes, while one strain carried the *seb* gene and six strains carried the *sec* gene.

**TABLE 6:** Genotyping of *S. aureus* enterotoxins along with their corresponding composition ratios^*a*^

Enterotoxin type	*S. aureus* (%)	MRSA (%)	MSSA (%)	*P*
*sea*	17 (16.3%)	2 (10.0%)	15 (17.9%)	*P* = 0.393
*seb*	9 (8.7%)	4 (20.0%)	5 (6.0%)	*P* = 0.045
*sec*	20 (19.2%)	6 (30.0%)	14 (16.7%)	*P* = 0.174
*sed*	0 (0.00%)	0 (0.0%)	0 (0.0%)	NA
*sea + seb*	1 (0.9%)	1 (5.0%)	0 (0.0%)	NA
*sea + sec*	2 (1.8%)	0 (0.0%)	2 (2.4%)	*P* = 0.486
*sec + sed*	2 (1.8%)	0 (0.0%)	2 (2.4%)	*P* = 0.486
*see*	0 (0.00%)	0 (0.0%)	0 (0.0%)	NA
Non-enterotoxin carrier	53 (51.0%)	7 (35.0%)	46 (54.8%)	*P* = 0.112
Total	104	20	84	

^*a*^ NA, not applicable; MRSA, methicillin-resistant *Staphylococcus aureus;* MSSA, methicillin-sensitive *Staphylococcus aureus.*

### SCCmec typing

SCCmec type IVa (target band size of SCCmec IVa: 776 bp) was found in 13 out of 20 (65%) MRSA strains. The remaining 7 MRSA strains could not be classified, which led to a classification as SCCmec type NT (Table 4).

### Multilocus sequence typing (MLST)

**Fig 1:**
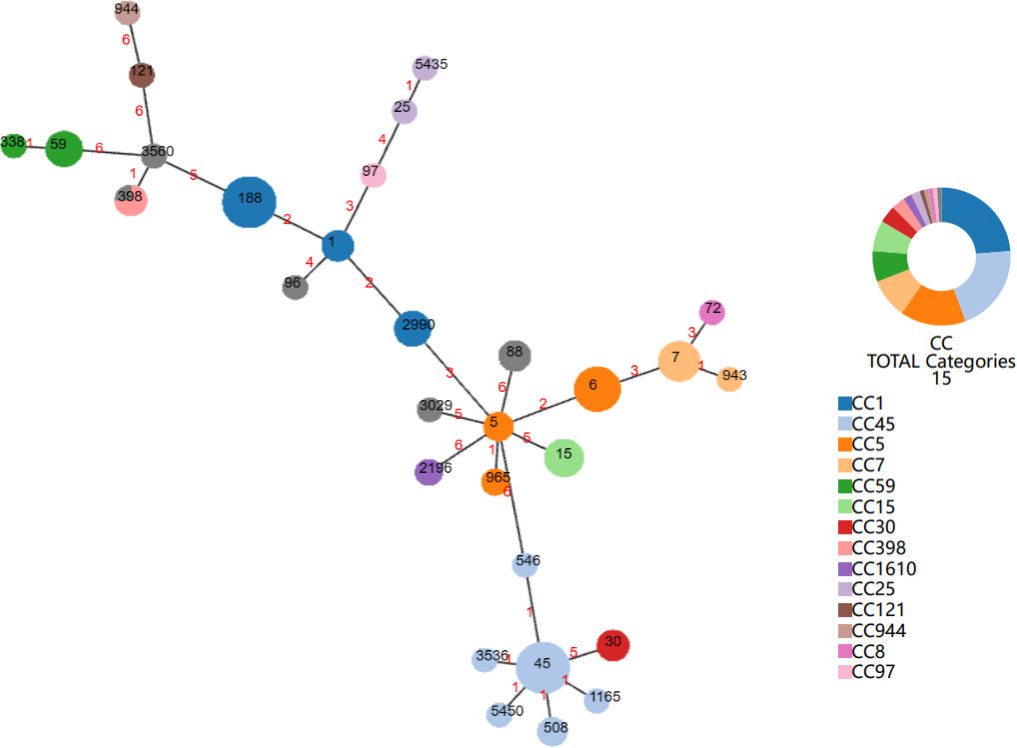
Minimum spanning tree based on the genotypic structure of all *Staphylococcus aureus* isolates.

All 104 *S*. *aureus* strains were characterized by MLST; the results are presented in Fig. 1. The predominant MLST types were ST45, ST188, and ST6, accounting for 12.5%, 12.5%, and 9.6% of the isolated *S. aureus* strains, respectively. Among MRSA strains, the common types were ST59, ST45, and ST1, representing 30.00%, 25.00%, and 10.00% of the isolated MRSA strains, respectively. In contrast, the main MLST types observed in MSSA were ST188, ST6, ST45, and ST7, constituting 15.48%, 10.71%, 9.52%, and 9.52% of the isolated MSSA strains, respectively (Fig. 1).

### Clonal diversity

As shown in Fig. 1, minimum spanning tree (MST) analysis was used to explore MLST typing in 104 strains of *S. aureus*, revealing their classification into 14 CCs. Notably, the predominant CCs were CC1, CC45, CC5, CC7, CC15, and CC59, constituting proportions of 22.1%, 19.2%, 14.4%, 8.6%, 6.7%, and 6.7%, respectively. Within the primary CCs, CC1 encompassed ST1, ST188, and ST2990, while CC45 predominantly featured ST45, ST508, ST546, ST1165, ST3536, and ST5450. MRSA primarily belonged to CC59 and CC45, each comprising 35.0% of the isolates. In contrast, MSSA predominantly exhibited CC1, CC5, and CC45, with proportions of 25.0%, 16.6%, and 15.4%, respectively.

### Association of enterotoxin genes with certain STs

Nine MLST types were selected to analyze the association between toxin genes and the ST types of *S. aureus*. All strains of ST6 and ST508 *S. aureus* expressed the sea gene. 66.67% of ST59 S. aureus expressed the *seb* gene, significantly higher than the other 8 ST types (*χ*² = 8.580, *P* = 0.0034). Additionally, 84.62% of ST45, 75.00% of ST30, and 66.67% of ST5 *S. aureus* strains expressed the sec gene. 66.67% of ST5 *S. aureus* strains expressed the sed gene, while strains of ST338 and ST59 *S. aureus* expressed the lukS and lukF-PV genes (Table S1).

### Association of antibiotic resistance with certain STs

**Fig 2:**
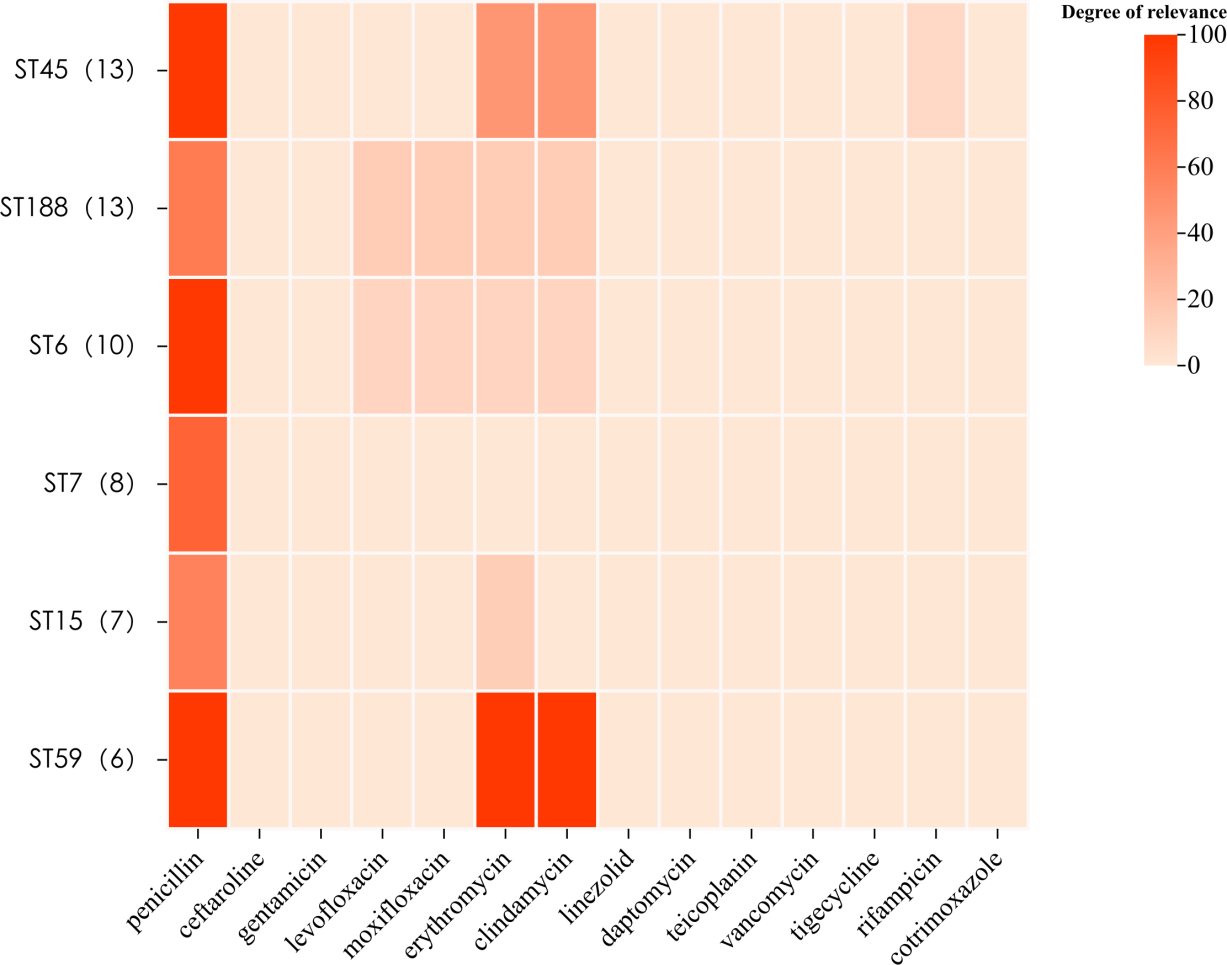
Heatmap of the association of MLST typing with antibiotic resistance.

We investigated the relationship between molecular typing and antibiotic resistance in 104 strains of S. *aureus* using six major MLST types. The results revealed a close association between ST45 and ST59 with MRSA strains, both exhibiting resistance to penicillin. Specifically, ST59 displayed a remarkably high resistance rate of 100% to erythromycin and clindamycin, significantly higher than the other five major ST types (for erythromycin resistance: *χ*² = 17.18, *P* < 0.0001; for clindamycin resistance: *χ*² = 18.78, *P* < 0.0001). Among these six types, only ST45 *S. aureus* strains showed resistance to levofloxacin (7.69%) (Figure 2).

## DISCUSSION

The aim of this study was to investigate and compare the molecular epidemiology and antibiotic resistance of *S. aureus* colonizing the intestinal tract of pediatric inpatients and outpatients of different age groups (9). *S. aureus* is a commensal pathogen that is responsible for a variety of nosocomial and community-acquired infections (19). Previous research primarily focused on *S. aureus* colonization of the nose and skin (20). However, there is evidence that the intestine, rather than the nose, is the primary site of colonization by *S. aureus* and may even serve as a reservoir (8, 21). It is known that colonization frequency can vary depending on geographical location and socioeconomic factors (22). Within the population we studied, we found a prevalence of 8.0% for intestinal colonization with *S. aureus* (8, 23). These findings are lower compared to a previous study carried out in Guangzhou, which found a prevalence of 20%. The prevalence of MRSA was also significantly higher in the Guangzhou study (4.5%) than in this study (1.5%) (8). In addition, the Guangzhou study found a positivity rate of 77.4% for *S. aureus* in patients diagnosed with gastroenteritis, while in this study only 44.2% of patients diagnosed with gastroenteritis were *S. aureus* positive. Moreover, we found that the prevalence of intestinal *S. aureus* carriage in children from different countries was between 20.8% and 29.1% (95% confidence interval [CI]) (24). This further demonstrates that the *S. aureus* carriage rates in our hospital are significantly lower compared to previous studies. Differences in the geographic distribution of patients seeking medical care and in the complexity of their illnesses, coupled with higher antibiotic exposure, may have contributed to the disparity. Additionally, age distribution could be a contributing factor, as previous studies have shown that newborns have a higher intestinal *S. aureus* carriage rate, reaching up to 73% and is consistent with our finding (24). In general, high population density and poor hygiene promote transmission of *S. aureus* (25). In addition, transmission to patients can occur through healthcare workers if they are themselves colonized or cause transmission between patients (26). The most effective measures to counteract this regular route of nosocomial transmission include regular and thorough hand disinfection and decontamination measures (27, 28).

It is worth noting that this study found the highest carriage rate of *S. aureus* in infants aged 29 days to 6 months (13.86%), which may be related to the incomplete development of immune function and lower immunity in this age group. In addition, this also indicates that most *S. aureus* strains do not necessarily originate from the hospital environment. For the age group of 6 months to 12 years, the intestinal carriage rate of *S. aureus* showed a negative correlation with increasing age. This is consistent with the findings of other studies, which indicate that the carriage rate in infants is highest within 6 months and gradually decreases with increasing age. Infants can acquire *S. aureus* from their mother. Chen et al. reported that women can transmit colonizing bacteria from the vagina to their infants at birth (29). In addition, there are reports suggesting that mothers can transmit MRSA to their infants through breastfeeding. Intestinal colonization with *S. aureus* is more common in breastfed infants than in bottle-fed infants (30). Colonization of hospitalized infants with *S. aureus* correlates with the length of hospital stay and the hospital environment (31). In this study, the colonization rate of *S. aureus* in infants aged 0–28 days was 3.92% and the colonization rate of MRSA was 0.49%. This indicated the trend of *S. aureus* colonization in the gut in the pediatric population.

*S. aureus* produces enterotoxins; these bacterial enterotoxins consist of five traditional genotypes: *sea*, *seb*, *sec*, *sed*, and *see* (32, 33). In this study, 104 *S*. *aureus* strains were subjected to enterotoxin genetic testing, which yielded a detection rate of 49.0% (51/104). Among them, *sec* had the highest detection rate at 23.1% (24/104), followed by *sea* at 19.2% (20/104). Notably, the *see* genotype was absent. These results are consistent with the study in Guangzhou (8), which also identified *sec* as the predominant enterotoxin in clinical *S. aureus* isolates. Previous studies have linked *sea* and *sed* to food poisoning caused by *S. aureus* (34). In this study, 7 of the 22 patients carrying *sea*-positive strains were diagnosed with enteritis, and 7 of the 22 patients carrying *sea*-positive strains were diagnosed with either bronchopneumonia or pneumonia, while the other four patients were diagnosed with other types of disease. These observations may suggest an association between *sea*-positive *S. aureus* and enteritis and pneumonia. We found that 83.65% of *S. aureus* isolates were resistant to penicillin, which is equivalent to the findings in Guangzhou (84.2%). We investigated the association between molecular typing and drug resistance of *S. aureus* strains for six major MLST types. ST45 and ST59 were closely associated with MRSA strains, and both of which showed penicillin resistance in all strains. Among them, ST59 had significantly higher resistance rates to erythromycin and clindamycin (100%) compared with the other five ST types, while being relatively more sensitive to levofloxacin, moxifloxacin, and linezolid. ST188 was predominantly associated with resistance to levofloxacin and moxifloxacin, while ST5 strains showed resistance to cotrimoxazole (trimethoprim/sulfamethoxazole).

A MST analysis was performed on MLST types and identified major CCs, including CC1, CC45, CC5, CC7, CC15, and CC59. These results are slightly different from those of the Guangzhou study (8), which mainly identified CC30, CC45, CC5, CC1, and CC15. There are also discrepancies in the major CC types isolated in studies in Germany and the United States (35–37), suggesting clonal diversity among *S. aureus* strains in different regions. This study found that both PVL-positive strains detected belonged to CC59, which is consistent with several reports linking PVL to CC59. In this study, the Seb gene was mainly expressed in CC59, with five out of seven CC59 strains carrying *sea. sec* expression was mainly observed in CC45, with 15 out of 20 CC45 strains carrying Sec, consistent with the toxin gene carriage patterns in MRSA isolated in a study from Taiwan.

Overall, it is important to find out how to reduce intestinal colonization with *S. aureus*. To date, there are no established routine methods for decolonization of the intestine. However, a recent clinical trial by Piewngam et al. showed that the use of probiotics can be effective in decolonizing *S. aureus* in the intestine (38). Further research is needed to translate these results into clinical practice.

Limitations of this study include its single-center design, which may not fully represent the pediatric population. In addition, the lack of multiple surveillance time points for hospitalized patients makes it difficult to distinguish between hospital-acquired and community-acquired *S. aureus*.

In conclusion, we found that the molecular characteristics of *S. aureus* in intestinal colonization of children have regional differences. To provide a theoretical basis for the prevention and control of *S. aureus* infections, increased surveillance of local *S. aureus* resistance and molecular epidemiological characteristics is needed.

## SUPPLEMENTAL MATERIAL

Table S1 spectrum.02394-24-s0001.csv Clinical and molecular information of all *Staphylococcus aureus* isolates in pediatric patients.

ASM does not own the copyrights to Supplemental Material that may be linked to, or accessed through, an article. The authors have granted ASM a non-exclusive, world-wide license to publish the Supplemental Material files. Please contact the corresponding author directly for reuse.
